# Sex matters: understanding wood–leaf hydraulic coordination in dioecious species in a drying world

**DOI:** 10.1093/treephys/tpaf133

**Published:** 2025-10-22

**Authors:** Chiara Amitrano, Angela Balzano, Riccardo Motti, Maks Merela, Veronica De Micco

**Affiliations:** Department of Agricultural Sciences, University of Naples Federico II, Piazza Carlo di Borbone 1, Portici (Naples), Italy; Department of Wood Science and Technology, Biotechnical Faculty, University of Ljubljana, Rožna Dolina,Cesta VIII/34, 1000 Ljubljana, Slovenia; Department of Agricultural Sciences, University of Naples Federico II, Piazza Carlo di Borbone 1, Portici (Naples), Italy; Department of Wood Science and Technology, Biotechnical Faculty, University of Ljubljana, Rožna Dolina,Cesta VIII/34, 1000 Ljubljana, Slovenia; Department of Agricultural Sciences, University of Naples Federico II, Piazza Carlo di Borbone 1, Portici (Naples), Italy

**Keywords:** dioecy, leaf–stem coordination, photosynthesis, quantitative wood anatomy, sexual dimorphism, SLA

## Abstract

Sexual dimorphism in dioecious species can shape divergent hydraulic strategies in response to environmental stress, yet integrative studies linking anatomical and physiological traits across different plant organs remain scarce. We investigated sex-specific water-use strategies in two Mediterranean shrubs, *Pistacia lentiscus* L. and *Rhamnus alaternus* L., by analyzing leaf and wood anatomy, leaf functional traits, gas exchange and chlorophyll fluorescence. Male plants of both species exhibited conservative morpho-anatomical traits, including smaller, thicker leaves, lower specific leaf area (SLA), higher dry matter content and reduced intercellular spaces, traits typically associated with drought resistance strategies. In *P. lentiscus*, these traits correlated with higher photosynthetic rates and F_v_/F_m_ values, alongside greater stomatal density and vessel frequency, suggesting coordinated investment in carbon gain and hydraulic efficiency/safety. Conversely, females displayed acquisitive traits (higher SLA, wider intercellular spaces, lower vessel frequency), potentially enhancing photosynthesis under mesic conditions but increasing vulnerability to drought-induced embolism. In *R. alaternus*, female individuals maintained higher net photosynthesis and instantaneous water- use efficiency, while males exhibited greater F_v_/F_m_ and a decoupled leaf–wood coordination. These findings suggest that males may adopt safer hydraulic architectures, while females, potentially constrained by reproductive demands, pursue efficiency-driven strategies, still maintaining vessel redundancy in wood. As aridity intensifies in Mediterranean regions, such dimorphism may influence population dynamics, sex ratios and species resilience. Our results underscore the ecological significance of species-specific sex-based hydraulic variation and the necessity of incorporating sex into trait-based models of plant responses to climate change.

## Introduction

Dioecious plants represent ~7.5% of flowering plants and about 6% of angiosperms (Tognetti 2012), playing a significant role across diverse terrestrial ecosystems. In these species, males and females have evolved distinct adaptive strategies to cope with environmental stressors, primarily due to difference in reproductive costs (Dawson and Ehleringer 1993). Indeed, in dioecious species, reproduction entails different energetic costs for the two sexes (Jönsson 2000). Females, given their role, typically allocate more resources for reproduction compared with males, since seed and fruit development is highly resource-demanding (Hultine et al. 2016). Males, on the other hand, allocate resources to flower and pollen production, which also demands energy, though typically not as much as fruit and seed production (Xia et al. 2020). Consequently, while both sexes reallocate resources from vegetative growth to reproduction, females often face higher energetic and structural costs, as shown in *Populus purdomii*, where female individuals require a longer time than males for leaves to photosynthesize enough to compensate for the energy invested in reproductive structures (Lei et al. 2017). This sexual divergence in resource allocation has made dioecious species ideal models for studying trade-offs between growth, reproduction and stress tolerance (Cepeda-Cornejo and Dirzo 2010). When resources are limited, greater allocation to reproduction must occur at the expense of vegetative growth. This trade-off likely contributes to the spatial segregation of sexes in natural populations, with females more often occupying resource-rich microsites and males persisting in more resource-limited environments, a phenomenon also contributing to sexual dimorphism (Shine 1989).

Indeed, over the past two decades, numerous studies have highlighted sex-based differences in photosynthetic efficiency (Dawson and Geber 1999), water-use (Rowland and Johnson 2001), plant phenology (Delph 1999), tolerance to herbivory (Cornelissen and Stiling 2005) and even antioxidant profile (Zhang et al. 2010). Such findings suggest the existence of sex-specific adaptive strategies under environmental stress conditions. In Mediterranean ecosystems, climate change is intensifying stress factors by altering temperature and precipitation patterns, leading to more frequent extreme weather events (Calvin et al. 2023). Understanding how males and females of dioecious species respond to these stresses is crucial for formulating effective conservation strategies and protecting the diversity and resilience of these plants. Previous studies, mostly conducted on *Populus* spp., highlighted that male individuals often exhibit greater tolerance to both biotic and abiotic stresses (Li et al. 2016, Xia et al. 2020). For instance, Zhang et al. (2012) observed that under drought stress, *Populus cathayana* males exhibited superior self-protection of the photosynthetic system, accumulated more osmotic compounds and maintained a more effective enzymatic detoxification cycle to mitigate the harmful effects of reactive oxygen species (ROS), compared with females. Similar results were found in *P. cathayana* and *Populus yunnanensis* by Xu et al. (2008) and Chen et al. (2010) in which, under drought stress, female plants had a more severe decrease in net photosynthesis compared with males. However, most studies have focused on physiological and biochemical traits, with little or no attention given to anatomical traits involved in acclimation strategies.

Plants respond to environmental changes to reduce stress levels and improve the absorption of scarce resources by modifying different aspects of their metabolism, also developing a completely different anatomical organization of their organs (Nicotra et al. 2010). There is common agreement that plant anatomy is ‘at the heart of modern botany’, playing a crucial role in understanding physiology and ecology, serving as a valuable tool for exploring plant hydraulic acclimation capacity and plasticity in response to climate change (Sokoloff et al. 2021). Indeed, modifications in morpho-anatomical traits, and their combination rather than individual features, are crucial for drought escaping or resistance strategies, as they determine the plant’s ability to improve water retention and hydraulic conductivity (De Micco and Aronne 2008, Gleason et al. 2016; Zahedi et al. 2025). In dry environments where water is the limiting resource, the correlation between wood and leaf anatomical traits associated with hydraulic transport can help predict plant adaptive strategies (Hacke et al. 2015). The development of peculiar anatomical features is part of the intraspecific genotypic and phenotypic variation that confers different degrees of plasticity in response to environmental changes. In dioecious species, such variation is also associated with sex (Olano et al. 2017). Despite this, very few studies so far have explored the hydraulic strategies, focusing on hydraulic coordination across tissues, in dioecious species and results suggest that males used more conservative (safety-prioritizing) strategies to favor survival (Tognetti 2012, Hultine et al. 2016). If so, increasing aridity in the Mediterranean environments may amplify performance differences between sexes, potentially leading to skewed sex ratios in natural populations, confirming what has already been found *Juniperus communis* subsp. *alpina* in Sierra Nevada (Spain) over 2000 m a.s.l.: a higher proportion of male individuals under higher elevations, thus higher environmental stress (Ortiz et al. 2002). To the best of our knowledge, there are few studies considering leaf anatomical development in dioecious species (McKown et al. 2017), and none considering the relations between wood and leaf anatomy to understand the different hydraulic strategies of male and female in Mediterranean shrubs.

In this study, we aim to obtain a deeper understanding of sex-specific hydraulic performances in two Mediterranean shrubs, *Pistacia lentiscus* L. and *Rhamnus alaternus* L., growing in southern Italy, by determining wood and leaf anatomical traits, as well as leaf eco-physiological traits related to water use and photosynthetic efficiency. Specifically, we address the following questions: (i) Do these species exhibit secondary sexual dimorphism in wood and leaf anatomy related to hydraulic function? (ii) How does sex influence hydraulic strategies in both xylem and leaf tissues? (iii) Do the two species adopt similar strategies? (iv) Are males better equipped with safety-oriented hydraulic mechanisms? Understanding these patterns is crucial for anticipating the ecological consequences of climate change on dioecious species and for guiding conservation efforts.

## Materials and methods

### Study site and plant material

**Figure 1:**
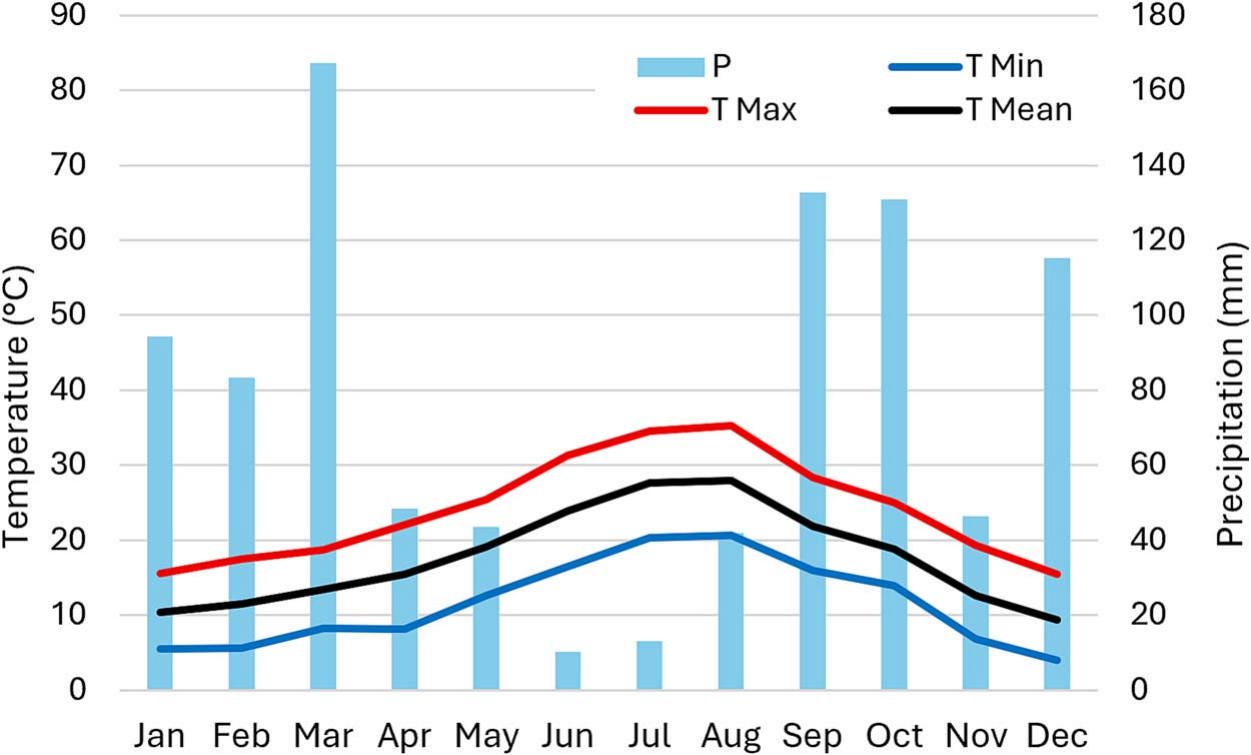
Climatic diagram of monthly average maximum (higher line), mean (medium line) and minimum (lower line) temperatures (T) and total monthly precipitation (P) for 2024, elaborated from the Casarea/Casalnuovo (IT) weather station data (https://agricoltura.regione.campania.it/meteo/meteo_2024.html, accessed date: 24 June 2025).

The study was conducted in the Gussone Park (40°48′52.9″N;14°20′54.8″E, 25 m a.s.l., 40.81469, 14.34857) at the premises of the Department of Agricultural Sciences of the University of Naples Federico II, in Portici (Naples, Southern Italy). The Gussone Park is an urban forest dominated by *Quercus ilex* L. The climate is Mediterranean, with hot, dry summers alternating with wet winters. Meteorological data of the year 2024 are from the weather station located at Casarea/Casalnuovo (Naples, Italy) (40°53′25.6″N;14°21′28.0″E, 25 m a.s.l.) within the Campania Region weather network (https://agricoltura.regione.campania.it/meteo/meteo_2024.html, accessed date: 24 June 2025) (Figure 1). The study was conducted on *P. lentiscus* and *Rhamnus alaternus* shrubs. *Pistacia lentiscu*s is an evergreen shrub or small tree that can reach up to 3–4 m in height. It has alternate, paripinnate leaves composed of 4–8 leathery leaflets. The flowers are small and arranged in axillary clusters. The fruits are globose drupes that turn from red to black as they mature. The bark is grayish and produces an aromatic resin known as mastic (Rivas-Martínez and Loidi 1999). *Rhamnus alaternus* is an evergreen shrub or small tree, usually growing up to 5 m tall. It has alternate, simple, leathery leaves, ovate to elliptical in shape, glossy on the upper surface, with margins entire or slightly toothed. The small yellowish-green flowers are typically clustered in the leaf axils. The fruit is a globose berry that ripens to a shiny black color and usually contains two or three seeds. The bark is smooth and gray-brown, pubescent on younger branches (Quézel and Médail 2003). Three female and three male individuals of *P. lentiscus* and of *R. alaternus* of similar size were selected and labeled during the flowering period in spring 2024 by identifying their reproductive structures. To minimize the influence of microsite variability, male and female plants of each species were co-existing. A limitation of this study is this relatively small sample size (*n* = 3 individuals per sex per species). This constraint was related to the availability of mature trees co-occurring under comparable microsite conditions. Each individual was in fact represented by multiple leaves samples, providing detailed trait estimates and enhancing the robustness of comparisons. While this restricted replication prevents broad generalizations, it ensured that sex-related differences could be interpreted without the confounding effect of strong micro-environmental heterogeneity.

### Ecological leaf functional traits

Leaf functional traits were measured on 10 leaves per plant per sex (30 leaves per sex) in both species following the method by Cornelissen et al. (2003). Leaves were collected in July and digital images of the leaf lamina were captured to calculate leaf area (LA) using the ImageJ software (ImageJ; Rasband, W.S., US NIH, Bethesda, MD, USA). Then, the fresh weight (FW) was recorded, and leaves were saturated in distilled water for 48 h in the dark. Saturated leaves were re-weighted to assess the saturated weight (SW). Lastly, the dry weight (DW) was obtained by oven-drying leaflets at 60 °C for 3 days, until they reached a constant weight. From these measurements the following parameters were calculated: (i) the specific leaf area (SLA), calculated as LA/DW, expressed in cm^2^ g^−1^, (ii) leaf dry matter content (LDMC), calculated as DW/SW and expressed as g g^−1^, (iii) the relative water content (RWC), calculated as RWC = (FW − DW)/(SW − DW), expressed as a percentage.

### Gas-exchange and chlorophyll fluorescence measurements

Gas-exchange and chlorophyll *a* fluorescence were performed twice in the periods of maximum vegetative activity corresponding to high levels of environmental stress (July 2024 and September 2024) on fully expanded leaves with the same exposition to sunlight within 2 h of solar noon (i.e. between 11:00 and 13:00 h). Gas-exchanges were measured with a portable gas exchange analyzer (LCA 4; ADC BioScientific Ltd, Hoddesdon, UK) equipped with a broad-leaf PLC (cuvette window area, 6.25 cm^2^). Ambient values for PAR, relative humidity and carbon dioxide concentration were used. The following parameters were then calculated: (i) Net CO_2_ assimilation rate (A_N_), expressed in μmol CO_2_ m^2^ s^−1^, (ii) transpiration rate (Tr), expressed in mmol H_2_O m^2^ s^−1^, (iii) stomatal conductance (g_S_), expressed in mol H_2_O m^2^ s^−1^ and (iv) instantaneous water-use efficiency (iWUE), calculated as A_N_/Tr.

On the same dates, on the same leaves, chlorophyll *a* fluorescence was performed both in the light and in the dark using a portable ‘plant stress kit’ instrument, which included a light-adapted Φ_PSII_ meter, a dark-adapted F_v_/F_m_ meter and 10 dark-adaptation leaf clips (Opti-Sciences Inc., Hudson, TX, USA). Light-adapted measurements were recorded by applying a saturating pulse to obtain the maximum light-adapted fluorescence (F_m_′) and steady-state fluorescence (F_s_). For dark-adapted measurements, leaves were adapted to darkness for 30 min, and then, measurements were recorded by applying a saturating pulse light to obtain the maximal fluorescence (F_m_) and minimal fluorescence (F_0_) values. The PSII maximum photochemical efficiency (F_v_/F_m_) was determined as F_v_/F_m_ = (F_m_ − F_0_)/F_m_. Following the method of Genty et al. (1989) the quantum yield of PSII electron transport (Φ_PSII_) was determined as Φ_PSII_ = (F_m_′ − F_s_)/F_m_′. Using the equation of Krall and Edwards (1992), the electron transport rate was calculated. Finally, the non-photochemical quenching (NPQ) was calculated as NPQ = (F_m_ − F_m_′)/F_m_′ following Bilger and Björkman (1991).

### Anatomical characterization of leaves

Five leaves per plant per plant sex per each species were sampled in July and stored in the FAA fixative solution (40% formaldehyde, glacial acetic acid, 50% ethanol, 5:5:90 by volume). Each leaf was dissected into lamina sub-samples of about 5 × 5 mm, dehydrated in an ethanol series (up to 95%), then infiltrated and embedded in the acrylic resin JB4® (Polysciences, Germany). Resin-embedded lamina samples were cut to obtain semi-thin cross-sections (5 μm thick), by means of a rotary microtome (Bio-optica, pfm medical 3004 M). The sections were stained with 0.5% Toluidine blue O in water (Feder and O’Brien 1968), and mounted on slides with mineral oil for microscopy. The sections were observed under a light microscope (BX51; Olympus) and images at 20× magnification were captured through a digital camera (Olympus EP50). The images were analyzed using Olympus CellSens standard software to quantify anatomical features. The thickness of both the adaxial and abaxial cuticles and epidermis, along with the palisade and spongy parenchyma, was measured in five distinct regions of the leaf lamina. To estimate the compactness of the palisade and spongy parenchyma, the proportion of tissue area occupied by intercellular spaces was assessed in three separate regions of the lamina, excluding veins and any areas with section defects to ensure data accuracy (De Micco et al. 2020).

Five leaves per plant per plant sex per each species were sampled in the same period to determine stomatal traits. The abaxial epidermis in the median region of each leaf lamina was peeled, avoiding the midrib as well as the margin. Stomatal size in terms of length (μm) of guard cells was measured in ten stomata per epidermal strip, with three strips analyzed per leaf. Furthermore, the stomatal density (number of stomata per mm^2^) was calculated in five regions per each leaf.

### Anatomical characterization of wood

Three disks per sex per species were collected at diameter at breast height from one of the main stems in April 2024. To avoid age effects, we selected stems with similar diameter. Tension wood was identified and traced on the disks and normal wood was selected for the analysis by making cuts along the opposite diagonal. This procedure ensured exclusion of tension wood bands, allowing analysis of growth rings extending from bark to pith in normal wood. The obtained samples were soaked in a solution composed of half 50% ethanol (diluted with water) and half glycerin to facilitate softening. Cross sections 15–20 μm thick were cut by using a Leica SM2010R sliding microtome (Leica Biosystems Nussloch GmbH, Nußloch, Germany). These sections were stained with a water-based solution of safranin and Astra blue (Van der Werf et al. 2007). Subsequently, the stained sections were permanently mounted on glass slides using Euparal mounting medium (Chroma 3C-239 Waldeck, Münster, Germany). The slides were examined under a Zeiss Axio Imager A.2 light microscope (Carl Zeiss Microscopy, White Plains, NY, USA), and images were captured with a Zeiss Axiocam 712 color camera (Carl Zeiss Microscopy GmbH, Jena, Germany). Quantitative wood anatomy analysis was performed using Olympus AnalySIS software version 3.2, aiming to quantify vessel lumen area (VLA) and vessel frequency in the most recent five complete growth rings beneath the bark for all trees (common across individuals). Each ring was sectioned horizontally into three equal parts (region of interest, ROI), and analyses were conducted independently on each ROI to increase the statistical robustness of the findings. During the automated quantification of VLA, a filtering threshold was applied to exclude cells smaller than the smallest vessel observed in the captured images. These smaller cells included fiber cells, imperforate tracheary elements and parenchyma cells, which are components of the ground tissue within the tree rings. The threshold for this filter was adjusted according to the specific section and species to ensure accuracy in distinguishing between vessels and the other cell elements. For vessel frequency, we counted the number of vessels solitary and in groups in a known area, according to Wheeler et al. (1989). Frequency distribution of the vessels in classes of lumen area was calculated by identifying classes with intervals of 200 –999 µm^2^ VLA, and classes with intervals of 2000 μm^2^ for VLA >1000 μm^2^.

**Table 1:** Leaf functional traits in terms of leaf area (LA), dry weight (DW), specific leaf area (SLA), leaf dry matter content (LDMC) and relative water content (RWC) in *P. lentiscus* and *R. alaternus* leaves from male (M) and female (F) plants.

	*P. lentiscus*			*R. alaternus*		
Parameters	M	F	*P*	M	F	*P*
LA (cm^2^)	2.63 ± 0.12	3.70 ± 0.25	^***^	10.57 ± 0.35	13.06 ± 0.47	^***^
DW (g)	0.05 ± 0.01	0.04 ± 0.01	NS	0.24 ± 0.05	0.15 ± 0.01	^*^
SLA (cm^2^ g^−1^)	58.68 ± 2.25	91.50 ± 6.93	^***^	80.59 ± 5.59	85.92 ± 1.34	^*^
LDMC (g g^−1^)	0.46 ± 0.01	0.41 ± 0.01	^***^	0.80 ± 0.20	0.38 ± 0.01	^*^
RWC (%)	83.63 ± 2.84	87.79 ± 0.97	NS	92.49 ± 0.98	91.55 ± 0.46	NS

Each value is the mean ± standard error (*n* = 30); *P*-values are reported as ^*^P < 0.05, ^***^P < 0.001 or non-significant (NS) according to an unpaired *t*-test.

### Data analysis

Statistical analyses were performed by means of SPSS 13 (IBM Corp., Armonk, NY, USA). Sex-based differences within each species were tested using unpaired *t*-test. Linear regression models were built in R software (version 4.4.3) to analyze the relationship between morpho-anatomical and eco-physiological data. Outliers were removed using the interquartile range method. Data, separated for males and females, were visualized using *ggplot2*, with regression lines and *R*^2^ annotations. To avoid pseudoreplication, the individual plant (*n* = 3 per sex per species) was considered the true unit of replication in all analyses. For each trait, mean values were calculated per individual and then used in statistical tests.

## Results

### Ecological leaf functional traits

Leaves of *P. lentiscus* and *R. alaternus* displayed similar trends in ecological leaf functional traits (Table 1). Male individuals of both species produced smaller leaves with lower SLA values (36% reduction in *P. lentiscus* and 6% in *R. alaternus*) but higher LDMC (15% increments in *P. lentiscus* and 112% in *R. alaternus*) compared with females. Significant differences in leaf dry weight (DW) were observed only in *R. alaternus*, where male leaves were 58% heavier than females. The RWC did not show significant differences between sexes in either species.

### Gas-exchange and photochemistry

As reported in Table 2, sex-related differences in eco-physiological performance were evident in *P. lentiscus*. Male individuals consistently exhibited higher photosynthetic rates in both July and September (17% and 24% increases, respectively) compared with females. Additionally, males had higher transpiration rates, with increases of 87% in July and 43% in September. Stomatal conductance was significantly higher in *P. lentiscus* males only in July with increments by 54%. In contrast, females exhibited in both monitoring periods, higher intrinsic water-use efficiency (iWUE). Regarding photochemistry, the only significant difference was in the F_v_/F_m_ ratio, where males showed once again higher values, with 5% and 11% increases in July and September, respectively.

**Table 2:** Leaf gas-exchange and photochemistry parameters in terms of net-assimilation rate (A_N_), transpiration rate (Tr), stomatal conductance (g_S_), instantaneous water-use efficiency (iWUE), maximum efficiency of PSII (F_v_/F_m_), the quantum yield of PSII electron transport (Φ_PSII_), electron transport rate (ETR) and non-photochemical quenching (NPQ) in *P. lentiscus* and *R. alaternus* leaves from male (M) and female (F) plants during the two monitoring periods (July and September 2024).

	**July 2024**			**September 2024**		
	M	F	*P*	M	F	*P*
***P. lentiscus***						
A_N_ (μmol CO_2_ m^2^ s^−1^)	14.08 ± 0.46	12.04 ± 0.23	^**^	16.10 ± 0.21	12.97 ± 0.19	^**^
Tr (mmol H_2_O m^2^ s^−1^)	0.58 ± 0.07	0.31 ± 0.05	^**^	0.56 ± 0.02	0.39 ± 0.04	^**^
g_S_ (mol H_2_O m^2^ s^−1^)	0.34 ± 0.06	0.22 ± 0.02	^*^	0.34 ± 0.06	0.23 ± 0.01	NS
iWUE (A_N_/Tr)	28.26 ± 4.31	45.41 ± 5.70	^*^	29.16 ± 0.21	36.37 ± 4.39	^*^
F_v_/F_m_	80.55 ± 0.26	76.73 ± 1.17	^**^	82.56 ± 1.25	74.55 ± 1.41	^**^
Φ_PSII_	72.93 ± 1.00	72.87 ± 0.70	NS	67.96 ± 1.83	74.82 ± 0.98	NS
ETR	344.4 ± 19.4	383.4 ± 29.8	NS	345.2 ± 16.18	384.3 ± 18.21	NS
NPQ	71.93 ± 1.00	71.87 ± 0.71	NS	66.96 ± 1.83	73.82 ± 0.98	NS
***R. alaternus***						
A_N_ (μmol CO_2_ m^2^ s^−1^)	9.46 ± 0.87	14.55 ± 1.30	^**^	10.80 ± 0.87	15.90 ± 1.30	^***^
Tr (mmol H_2_O m^2^ s^−1^)	1.39 ± 0.10	1.65 ± 0.10	NS	1.79 ± 0.01	1.81 ± 0.01	NS
g_S_ (mol H_2_O m^2^ s^−1^)	0.11 ± 0.01	0.13 ± 0.01	NS	0.45 ± 0.01	0.47 ± 0.01	NS
iWUE (A_N_/Tr)	6.72 ± 0.21	8.77 ± 0.35	^***^	6.01 ± 0.44	8.80 ± 0.69	^***^
F_v_/F_m_	0.81 ± 0.01	0.73 ± 0.01	^***^	0.84 ± 0.01	0.74 ± 0.01	^***^
Φ_PSII_	74.11 ± 1.00	71.12 ± 0.70	^*^	75.75 ± 1.00	72.77 ± 0.70	^*^
ETR	381.6 ± 29.8	345.6 ± 19.4	NS	363.4 ± 26.89	347.22 ± 19.44	NS
NPQ	70.12 ± 0.70	73.11 ± 1.00	^*^	71.77 ± 0.70	74.75 ± 0.74	^*^

Each value is the mean ± standard error (*n* = 5); *P*-values are reported as ^*^P < 0.05, ^**^P < 0.01, ^***^P < 0.001 or non-significant (NS) according to unpaired *t*-test.

**Figure 2:**
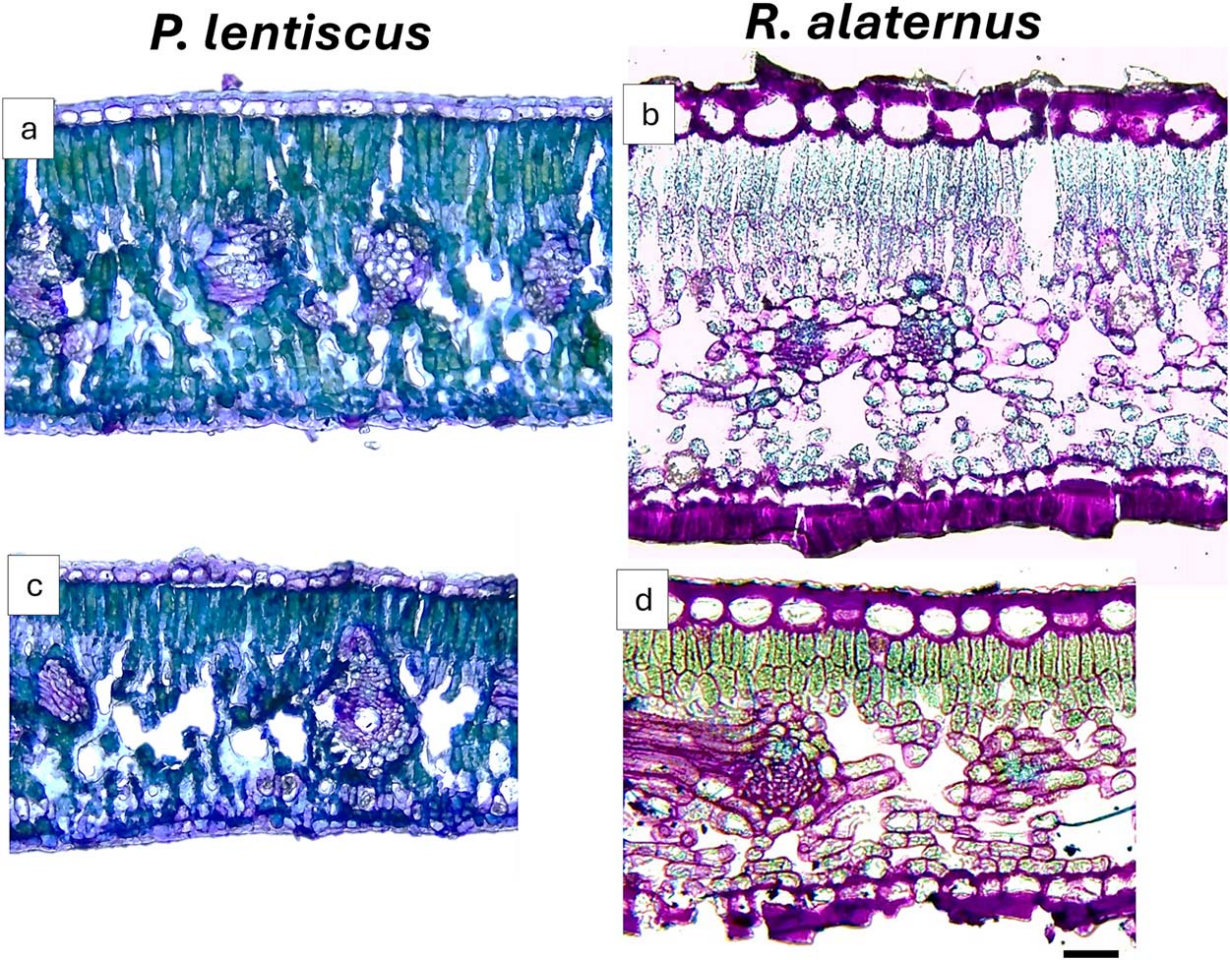
Light microscopy cross-sections of *P. lentiscus* (a, c) and *R. alaternus* (b, d) leaf lamina from male (a, b) and female (c, d) plants. Images are at the same magnification. Scale bar is on figure (d) and corresponds to 50 μm.

In *R. alaternus*, sex-related differences in eco-physiological performance were also observed (Table 2). Female individuals had higher photosynthetic rates in both seasons, with 35% and 32% increases in July and September, respectively, compared with males. Female plants also exhibited higher iWUE values by 23% and 46% during these months. In terms of photochemistry, males had higher F_v_/F_m_ ratios and Φ_PSII_ values in both seasons, with 10% and 13% increases in F_v_/F_m_ and 5% increases in Φ_PSII_ compared with females. However, females showed an increase in NPQ during both monitoring periods, with a 4% increment compared with males.

### Plant anatomy

#### Leaf lamina

In both species (Figure 2), male individuals developed thicker lamina (TT) due to increased thickness of upper and lower cuticle (UCT, LCT), upper epidermis (UET), palisade (PT) and spongy parenchyma (ST) compared with females (Table 3). The lower epidermis thickness (LET) was significantly higher only in males of *R. alaternus*, with 7% increases compared with females. The total leaf thickness (TT) in males was 19% and 10% higher than in females in *P. lentiscus* and *R. alaternus*, respectively. In contrast, the intercellular space percentage decreased in males of both species, showing reductions of 24% in *P. lentiscus* and 25% in *R. alaternus*. Regarding stomatal traits, male *P. lentiscus* individuals exhibited higher stomatal density (SD) but shorter stomatal length (SL) compared with females. In *R. alaternus*, males had lower stomatal density compared with females, while stomatal length did not differ significantly between sexes.

**Table 3:** Leaf anatomical parameters in terms of upper cuticle thickness (UET), upper epidermis thickness (UET), palisade thickness (PT), spongy thickness (ST), lower epidermis thickness (LET), lower cuticle thickness (LCT), intercellular spaces (IS), total thickness (TT), stomatal density (SD) and stomatal guard cell length (SL) in leaves of *P. lentiscus* and *R. alaternus* male (M) and female (F) plants.

	*P. lentiscus*			*R. alaternus*		
	M	F	*P*	M	F	*P*
UCT (μm)	9.47 ± 0.36	7.36 ± 0.20	^***^	15.59 ± 0.22	10.61 ± 0.11	^***^
UET (μm)	10.78 ± 0.24	9.62 ± 0.22	^*^	14.10 ± 0.18	13.51 ± 0.18	^*^
PT (μm)	96.48 ± 3.15	83.34 ± 2.08	^***^	63.31 ± 1.13	49.93 ± 0.54	^***^
ST (μm)	160.7 ± 3.96	131.2 ± 2.60	^*^	101.14 ± 1.16	97.55 ± 0.81	^*^
LET (μm)	7.88 ± 0.32	7.95 ± 0.37	NS	11.12 ± 0.11	10.43 ± 0.12	^***^
LCT (μm)	7.12 ± 0.35	5.70 ± 0.34	^***^	17.69 ± 11.43	11.43 ± 0.13	^***^
IS (%)	60.14 ± 0.52	79.38 ± 0.37	^***^	55.74 ± 0.52	74.98 ± 0.37	^***^
TT (μm)	291.7 ± 6.34	245.2 ± 2.74	^***^	228.4 ± 2.27	208.9 ± 1.12	^***^
SD (n mm^−2^)	110.1 ± 2.09	79.64 ± 15.34	^*^	71.09 ± 18.68	126.0 ± 2.09	^**^
SL (μm)	29.64 ± 0.92	34.00 ± 0.34	^***^	32.64 ± 0.92	32.70 ± 1.05	NS

Each value is the mean ± standard error; *P*-values are reported as ^*^P < 0.05, ^**^P < 0.01, ^***^P < 0.001 or non-significant (NS) according to unpaired *t*-test.

#### Wood anatomy

The wood of *P. lentiscus* and *R. alaternus* displayed distinct anatomical traits consistent with the features defined in the IAWA List of Microscopic Features for Hardwood Identification (Wheeler et al. 1989).

**Figure 3:**
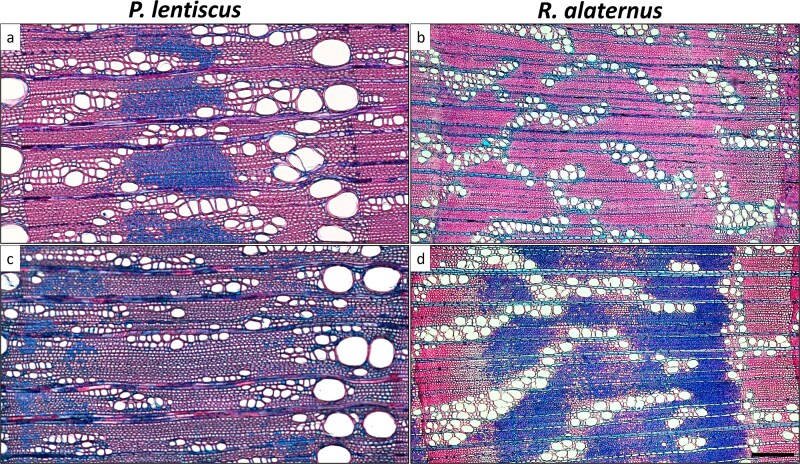
Light microscopy cross-sections of wood rings for male (a, b) and female (c, d) of *P. lentiscus* (a, c) and *R. alaternus* (b, d). Images are at the same magnification. Scale bar corresponds to 200 μm.

*Pistacia lentiscus* exhibited a ring-porous to semi-ring-porous structure (Figure 3a and c), with earlywood marked by large solitary vessels and latewood consisting of narrower, more densely packed vessels. Fibers appeared as from thin- to very-thick wall. Growth ring boundaries were distinct, demarcated by abrupt changes in vessel diameter. In contrast, *R. alaternus* displayed a diffuse-porous structure, with vessels distributed more uniformly across the growth ring (Figure 3b and d). Vessels occurred solitarily, in short radial multiples and in clusters. Vessel diameter was variable, and no sharp transition between earlywood and latewood was visible. Fibers were similar to those of *P. lentiscus*. Growth ring boundaries were less distinct than in *P. lentiscus*.

**Figure 4:**
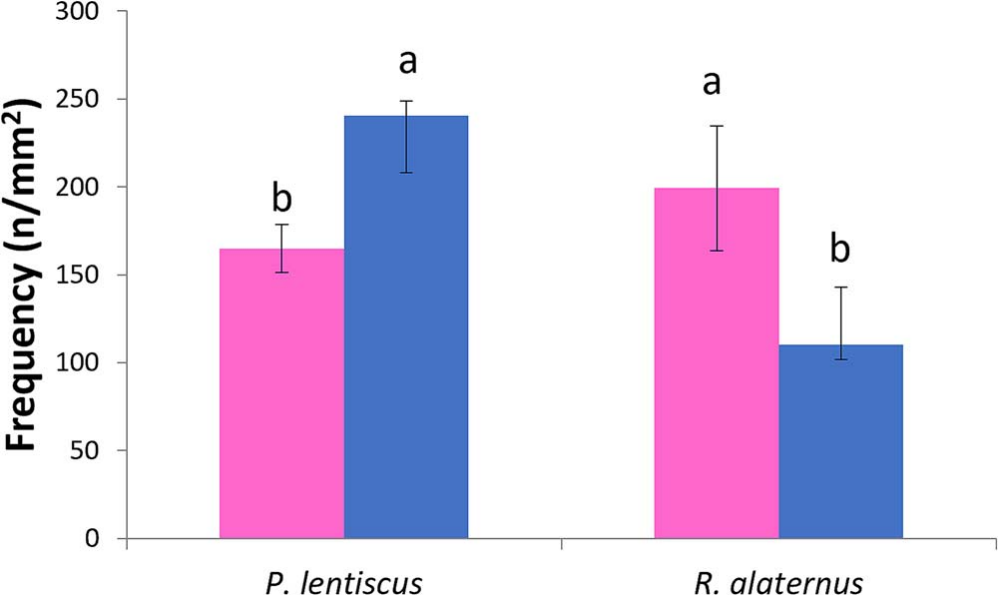
Vessel frequency (n mm^−2^) in the xylem of male (right) and female (left) individuals *P. lentiscus* and *R. alaternus*. Mean values and standard errors are shown. Different letters indicate statistically significant differences (*P* < 0.05).

**Figure 5:**
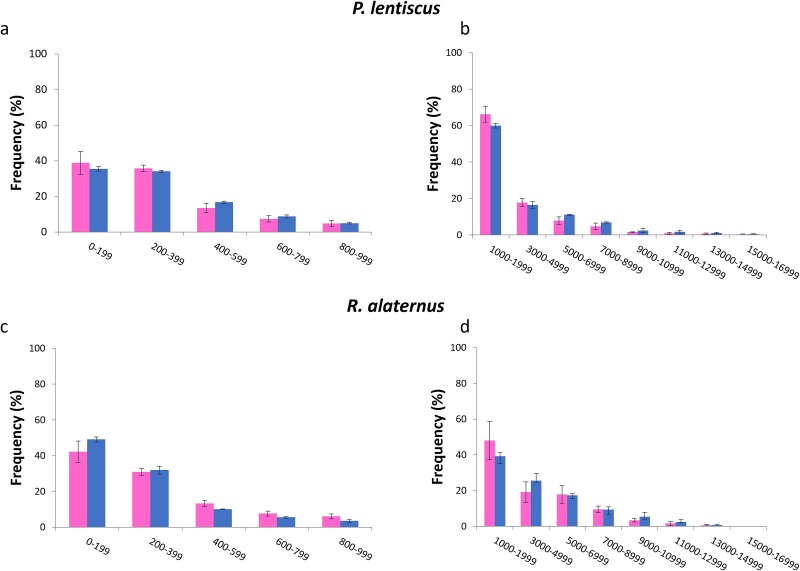
Distribution of vessels in classes of vessel lumen area (VLA) (μm^2^) in male (right) and female (left) individuals of *P. lentiscus* and *R. alaternus*, divided into two ranges: 0–999 (left panels) and 1000–16,999 (right panels). Bars represent mean vessel frequency per class ± standard error. None of the differences between males and females per each VLA class is significant.

Vessel frequency (Figure 4) showed a pronounced sexual dimorphism in *P. lentiscus*, where male individuals showed a significantly higher vessel frequency than females. In *R. alaternus*, females showed vessel frequency significantly higher than males. The distribution of VLA (Figure 5) was concentrated in the classes 0–199 μm^2^ and 200–399 μm^2^ in both species, with a strong decrease in the number of vessels in higher diameter classes, confirming the anatomical dominance of narrow to intermediate vessels in these Mediterranean shrubs. Vessels with a diameter of more than 2000 μm^2^ were uncommon in both species and sexes. In *P. lentiscus*, male individuals showed a tendency toward higher vessel area values in the intermediate classes, especially in 400–599, although these differences were not statistically significant. In *P. lentiscus*, female showed slightly higher frequencies in the lower diameter classes, while males showed higher values in 600–799, but differences were not significant.

#### Linear regression

**Figure 6:**
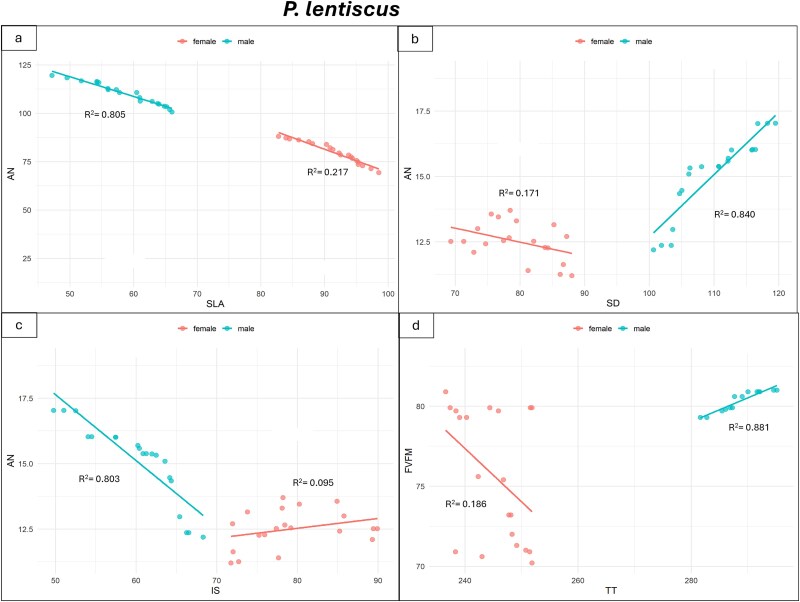
Linear regression between net-photosynthesis (A_N_) and specific leaf area (SLA) (a), net-photosynthesis (A_N_) and stomatal density (SD) (b), net-photosynthesis (A_N_) and intercellular spaces (IS) (c), and PSII maximum photochemical efficiency (F_v_/F_m_) and total thickness of leaf lamina (TT) (d) in *P. lentiscus* male (blue points) and female (pink points) individuals. *R*^2^ are shown. The *P*-values and statistical significance of linear regressions are shown in Table S1 available as Supplementary Data at *Tree*  *Physiology* Online.

**Figure 7:**
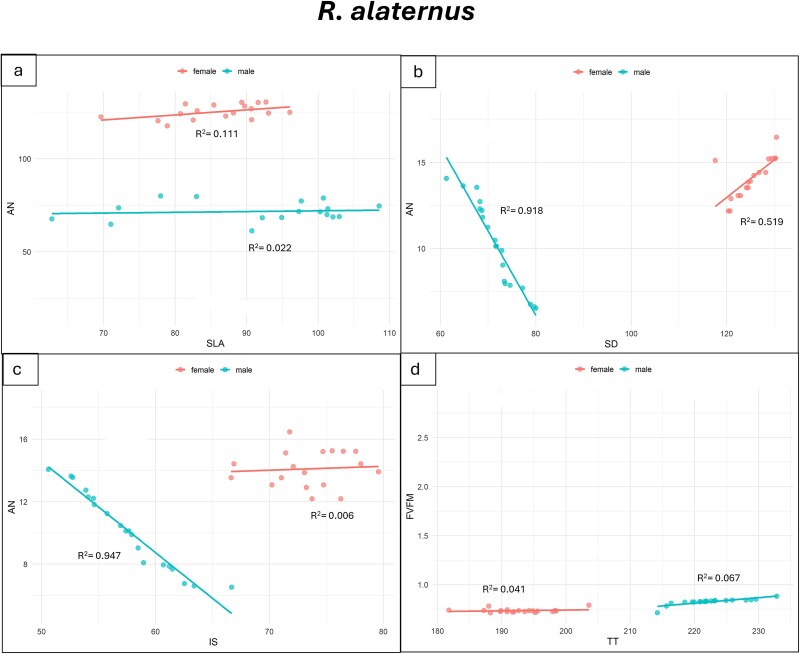
Linear regression between net-photosynthesis (A_N_) and specific leaf area (SLA) (a), net-photosynthesis (A_N_) and stomatal density (SD) (b), net-photosynthesis (A_N_) and intercellular spaces (IS) (c), and PSII maximum photochemical efficiency (F_v_/F_m_) and total thickness of leaf lamina (TT) (d) in *R. alaternus* male and female individuals. R^2^ are shown. The *P*-values and statistical significance of linear regressions are shown in Table S1 available as Supplementary Data at *Tree*  *Physiology* Online.

Significant relations were found between morpho-anatomical and eco-physiological traits in male and female individuals of *P. lentiscus* and *R. alaternus* (Figures 6 and 7). In *P. lentiscus* (Figure 6) males exhibited a significant negative relation between net-photosynthesis (A_N_) and SLA (*R*^2^ = 0.81; Figure 6a; Table S1 available as Supplementary Data at *Tree*  *Physiology* Online), and between A_N_ and intercellular spaces percentage (IS) (*R*^2^ = 0.80; Figure 6c; Table S1 available as Supplementary Data at *Tree*  *Physiology* Online). Differently, a significant positive relation was found between A_N_ and stomatal density (SD) (*R*^2^ = 0.84; Figure 6b; Table S1 available as Supplementary Data at *Tree*  *Physiology* Online), and PSII maximum photochemical efficiency (F_v_/F_m_) and total thickness of leaf lamina (TT) (*R*^2^ = 0.88; Figure 6d; Table S1 available as Supplementary Data at *Tree*  *Physiology* Online). Females presented weak negative relations for A_N_ − SLA (*R*^2^ = 0.22), F_v_/F_m_ − TT (*R*^2^ = 0.18) and A_N_ − SD (*R*^2^ = 0.17), and a weak positive relation for A_N_ − IS (*R*^2^ = 0.09).

In *R. alaternus* (Figure 7; Table S1 available as Supplementary Data at *Tree*  *Physiology* Online), males displayed a strong significant negative relation between A_N_ and SD (*R*^2^ = 0.92), and between A_N_ and IS (*R*^2^ = 0.95) (Figure 7b and c; Table S1 available as Supplementary Data at *Tree*  *Physiology* Online). In contrast, female showed a positive relation only between A_N_ and SD (*R*^2^ = 0.52), while no relation was observed between A_N_ and IS (*R*^2^ = 0.006). No relation was detected between A_N_ and SLA in either males (*R*^2^ = 0.02) or females (*R*^2^ = 0.11; Figure 7a; Table S1 available as Supplementary Data at *Tree*  *Physiology* Online). Additionally, females showed no relationship between F_V_/F_M_ and TT (*R*^2^ = 0.04; Figure 7d; Table S1 available as Supplementary Data at *Tree*  *Physiology* Online), whereas males exhibited a positive relationship between F_V_/F_M_ and TT (*R*^2^ = 0.67).

**Figure 8:**
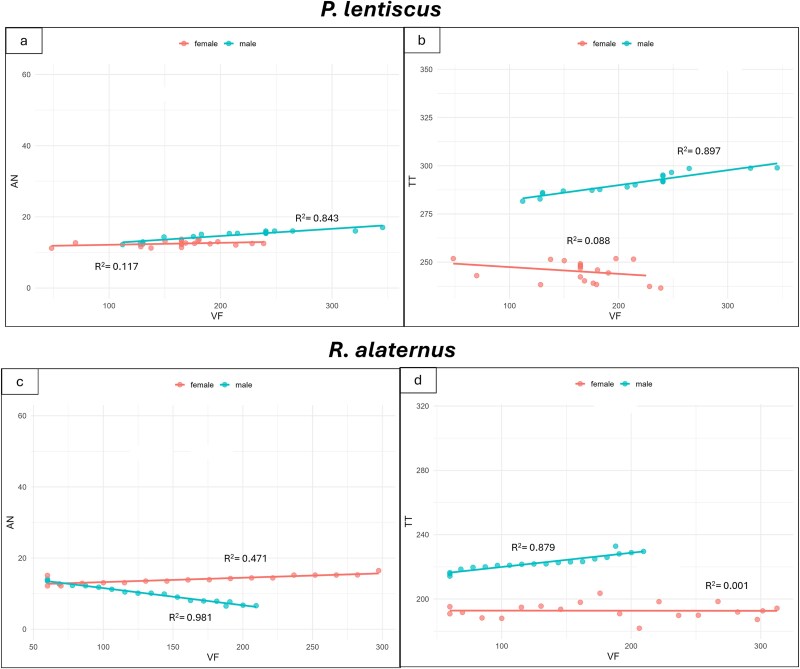
Linear regression between net-photosynthesis (A_N_) and vessel frequency (VF) (a, c), total thickness of leaf lamina (TT) and vessel frequency (VF) (b, d), in *P. lentiscus* (a, b) and *R. alaternus* (c, d) male and female individuals. *R*^2^ are shown. The *P*-values and statistical significance of linear regressions are shown in Table S1 available as Supplementary Data at *Tree*  *Physiology* Online.

To further explore wood–leaf functional coordination, correlations were also tested between vessel frequency (VF), net photosynthesis (AN) and lamina thickness (TT). In *P. lentiscus*, vessel frequency was positively related to both AN (*R*^2^ = 0.84; Figure 8a; Table S1 available as Supplementary Data at *Tree*  *Physiology* Online) and TT (*R*^2^ = 0.89; Figure 8b; Table S1 available as Supplementary Data at *Tree*  *Physiology* Online) in male individuals. In females, however, these relationships were weak or absent (*R*^2^ = 0.12 for AN and 0.09 for TT), suggesting a decoupling between vascular structure and functional performance. In *R. alaternus*, males exhibited an inverse relationship between VF and AN (*R*^2^ = 0.98; Figure 8c). As for TT, only males showed a positive correlation with VF (*R*^2^ = 0.88; Figure 8d; Table S1 available as Supplementary Data at *Tree*  *Physiology* Online), whereas in females no relationship was detected (*R*^2^ = 0.00).

## Discussion

This study provides new insights into sex-specific hydraulic strategies in two Mediterranean dioecious shrubs, *P. lentiscus* and *R. alaternus*. By integrating anatomical traits of wood and leaves, and morpho-physiological traits of leaves, we found that male and female individuals of these species exhibit a clear sexual dimorphism and distinct adaptive strategies. These results underscore the importance of sex in modulating water-use strategies and hydraulic architecture in Mediterranean woody species.

### Hydraulic and photosynthetic coordination in leaves

Male individuals exhibited leaf traits consistent with a conservative water-use strategy: smaller and thicker leaves, lower SLA, higher LDMC and reduced intercellular spaces. These features are typically associated with drought-adapted morphotypes that favor carbon retention and dehydration delay (Wright et al. 2004, Poorter et al. 2009). Increased mesophyll thickness in males likely supports more robust photosynthetic tissues and structural investment, in line with the observed higher F_v_/F_m_ in both species and increased photosynthetic rates in *P. lentiscus*. Similar patterns have been reported in dioecious species such as *P. cathayana*, where males exhibited greater photosynthetic performance under water stress, possibly due to denser foliage and more active stomatal control (Zhang et al. 2012, Li et al. 2016). In both species, male individuals exhibited increased thickness of the palisade and spongy mesophyll, as well as the upper epidermis and cuticle, compared with females. However, this anatomical reinforcement was accompanied by enhanced net photosynthesis only in *P. lentiscus*, while in *R. alaternus* it was associated solely with a higher F_v_/F_m_ ratio. This discrepancy may reflect species-specific differences in the coordination between structural and physiological leaf traits. In *P. lentiscus*, greater anatomical investment likely supports improved carbon assimilation capacity, possibly facilitated by higher stomatal density and mesophyll conductance (Brodribb et al. 2007, Flexas et al. 2012). Conversely, in *R. alaternus*, photochemical efficiency may improve in male individuals without a parallel increase in photosynthetic rates, potentially due to constraints in stomatal control or hydraulic efficiency (Sack and Scoffoni 2013, Anderegg et al. 2016). These findings support the idea that similar anatomical modifications can underpin divergent functional outcomes across species, depending on the degree of integration among photosynthetic, anatomical and hydraulic traits (Niinemets 2001, Carins Murphy et al. 2014).

The positive correlations observed between photosynthesis and stomatal density in *P. lentiscus* male individuals further confirm a functional coordination that optimizes gas-exchanges while minimizing hydraulic risk (Brodribb and Jordan 2008). This is further supported by reduced stomata size that would favor more prompts control of guard cell movements (Lawson and Matthews 2020). In contrast, *R. alaternus* males showed a different coordination: photosynthesis declined as stomatal density decreased, suggesting that their strategy may rely less on stomatal control (indeed, stomata size did not vary among sexes) and more on intrinsic anatomical or biochemical limitations (Flexas et al. 2012). Moreover, the weak correlation between net photosynthesis and SLA observed in *R. alaternus* suggests that leaf morphological traits are not primary drivers of photosynthetic capacity in this species. This may reflect a strategy where physiological performance is decoupled from structural efficiency, potentially favoring stress tolerance over carbon gain (Niinemets 2001, Poorter et al. 2009).

Female individuals of both species consistently maintained higher SLA and larger intercellular spaces compared with males. These traits are classically associated with acquisitive resource-use strategies, enabling higher relative growth rates, enhanced light capture and more efficient internal CO₂ diffusion (Wright et al. 2004). In particular, a high SLA typically reflects thinner leaves with lower tissue density, which reduces CO_2_ diffusion resistance and facilitates faster photosynthetic responses under favorable conditions (Shipley et al. 2006, Poorter et al. 2009).

However, these advantages may come at a cost: high SLA and abundant intercellular spaces are structurally linked to reduced mechanical strength and lower drought tolerance (Markesteijn et al. 2011, John et al. 2017). Leaf tissues with loose packing and low dry matter investment are more prone to turgor loss, cavitation susceptibility and thermal or oxidative damage under stress (Niinemets and Valladares 2006). Indeed, in tropical tree species, high SLA and low tissue density have been associated with higher mortality risk during drought events (Markesteijn et al. 2011). In our study, such traits in females suggest a prioritization of carbon assimilation and growth, potentially in relation to their greater reproductive investment (Obeso 2002). This could make females more productive under favorable mesic conditions but also more vulnerable under water-limited environments, a pattern consistent with sexual dimorphism observed in other dioecious species such as *Baccharis* (Dawson and Ehleringer 1993), *Populus* (Chen et al. 2014) and *Pistacia chinensis* (Karimi and Nasrolahpour-Moghadam 2016). However, evidence from the literature indicates that differences in water-use efficiency between sexes of woody plants are highly context-dependent and species-specific, often influenced by local water availability. For instance, males exhibited higher water-use efficiency than females in *Populus euphratica* under drought and salinity stress (Yu et al. 2023), as well as in *Ilex aquifolium* in north-western Spain (Sánchez Vilas et al. 2025), in line with expectations of higher female reproductive costs (Rozas et al. 2009). In contrast, other studies found males using water less conservatively than females (Cai et al. 2022). Still, no sex-related differences were detected in several species (McKown et al. 2017, Garcia-Barreda et al. 2022). This variability highlights that sex-specific water-use strategies cannot be generalized across dioecious woody plants but rather emerge from the interaction between species traits and environmental context.

Overall, these findings support the idea that there is a strong sexual dimorphism in leaf anatomical traits that is not merely structural, but reflects a deeper divergence in functional strategies. Female plants may adopt a more ‘opportunistic’ photosynthetic architecture, whereas males tend toward more conservative and drought-resilient morphologies, consistent with their generally lower reproductive cost and different ecological priorities.

### Xylem anatomy and species-specific hydraulic acclimation

Sexual dimorphism in wood anatomy was evident in vessel frequency. In *P. lentiscus*, males exhibited a higher vessel frequency compared with females, in line with their higher photosynthetic rates, and increased mesophyll and epidermal thickness. This suggests a coordinated enhancement of hydraulic conductivity and carbon assimilation capacity, possibly supporting greater productivity in male individuals. Such trait coordination between stem and leaf levels has been observed in species adapted to arid environments, where maintaining efficient, yet safe, water transport is essential (Brodribb and Holbrook 2003, Sperry et al. 2008). This is confirmed by correlations between wood and leaf functional traits in *P. lentiscus*, where male individuals exhibited strong positive relationships between vessel frequency and both net photosynthesis and lamina thickness (*R*^2^ = 0.84 and 0.89, respectively). Conversely, female individuals of *R. alaternus* exhibited foliar traits typically associated with more acquisitive water-use strategies—such as higher SLA, lower LDMC and reduced tissue thickness—features generally linked to lower structural resistance to dehydration. However, at the xylem level, females showed a higher vessel frequency compared with males. Although vessel size distribution did not differ significantly, this increased frequency may reflect a form of hydraulic redundancy that enhances overall safety by maintaining water transport even under partial embolism conditions (De Micco and Aronne 2008). In this sense, females may compensate for reduced leaf-level safety through greater xylem redundancy, supporting a mixed strategy that balances efficiency and resilience. This apparent decoupling between leaf and stem-level strategies may reflect a differential responsiveness of plant organs: while leaves are short-lived, photosynthetically active and highly plastic (Nicotra et al. 2010), xylem anatomy is developmentally fixed and may retain a more phylogenetically conserved or niche-encoded signal (Lachenbruch and McCulloh 2014, Lens et al. 2022). Such organ-level divergence has been noted in studies comparing hydraulic adjustment across tissues (Martínez-Cabrera et al. 2009), and may reflect an evolutionary optimization in which leaves respond rapidly to seasonal water stress, while stems maintain structural consistency and long-term conductivity. This interpretation is further supported by the contrasting correlations observed in male individuals: vessel frequency was strongly and positively related to lamina thickness (*R*^2^ = 0.88), but showed a markedly negative correlation with photosynthetic rate (*R*^2^ = 0.98), suggesting that greater investment in vessel formation may not correspond to increased gas exchange efficiency. Such a pattern may reflect a stress-compensatory anatomical strategy, where thicker leaves and reduced stomatal conductance help mitigate the risk of hydraulic failure associated with lower vessel redundancy. Conversely, females showed a moderate positive correlation between vessel frequency and photosynthesis, but no relationship with lamina thickness, pointing to a partially coordinated strategy less clearly aligned with xylem structure.

Although vessel frequency revealed clearer sex-based differences, the analysis of VLA classes did not show significant divergence between sexes in either species. In both *P. lentiscus* and *R. alaternus*, the majority of vessels were concentrated in the 200–799 μm^2^ range, with few conduits exceeding 1000 μm^2^, an anatomical pattern consistent with Mediterranean shrubs (Rosell et al. 2017). These results suggest that neither sex invests preferentially in markedly larger conduits, and that functional differences in xylem are more likely driven by vessel frequency (i.e. redundancy) rather than size. While the data do not support sex-specific investment in larger vessels, it is useful to consider these findings in the broader context of hydraulic function. From a physiological perspective, larger vessels are generally associated with increased hydraulic conductivity (Tyree and Zimmermann 2002), but also with greater vulnerability to xylem embolism under drought (Hacke et al. 2001), highlighting a classic trade-off between efficiency and safety. In dioecious species, such trade-offs may be modulated by sex-specific reproductive investment. While our data do not support a consistent pattern of wider vessels in females, the combination of foliar traits, such as higher SLA and lower tissue density, may reflect a more resource-acquisitive strategy, which could be advantageous in environments with intermittent water availability.

### Ecological implications

In this study ecophysiological measurements were confined to a single part of the growing season (July–September 2024), corresponding to the period of highest environmental stress. Because wood anatomy was assessed over the last five complete rings, whereas leaf traits refer only to 2024, wood–leaf comparisons should be interpreted as evidence of structure–function coordination rather than strict same-year matching.

As woody plant performance is influenced by intra- and inter-annual climatic variability, the results should be interpreted cautiously, as they may partly reflect responses to the exceptional conditions of that year.

Despite these limitations, the distinct hydraulic and photosynthetic strategies observed between sexes and species in this study have important ecological implications, particularly in the context of increasing climatic aridity across the Mediterranean region. Climate change is expected to intensify water stress through prolonged droughts, higher vapor pressure deficits and more frequent heatwaves (IPCC 2023). Under these conditions, individuals that maintain structurally reinforced leaves, reduced SLA and higher tissue density, like the male individuals in both *P. lentiscus* and *R. alaternus* may be better equipped to tolerate dehydration and sustain basic physiological function. These traits align with a ‘drought-avoidance’ strategy, minimizing water loss and safeguarding tissue integrity during dry periods (Choat et al. 2012, Bartlett et al. 2016).

Conversely, female individuals, characterized by thinner leaves, higher SLA and larger intercellular spaces, may experience greater water loss and tissue damage under stress, despite their potential advantage in carbon assimilation under mesic or transiently favorable conditions.

In *P. lentiscus*, this vulnerability seems not to be compensated by any sign of increased safety at the stem wood level. *In R. alaternus*, where females exhibit significantly higher vessel frequency but no difference in vessel size, greater xylem redundancy could enhance resistance to embolism, also conferring coordinated benefits in photosynthetic performance. This divergence suggests that species-specific anatomical syndromes modulate how sexual dimorphism translates into hydraulic risk. Thus, these findings suggest that, under increasing drought conditions, female individuals of *R. alaternus* may exhibit a lower vulnerability compared with those of *P. lentiscus.* The coordination observed in male individuals between leaf and xylem traits likely represents a more integrated adaptation to drought-prone environments, potentially conferring greater resilience under future climate scenarios.

## Conclusions

While the relatively small sample size limits broad ecological inferences, the convergence of anatomical and physiological evidence across traits and species supports the reliability of the main trends identified. These findings therefore offer valuable insights into sex-specific hydraulic strategies, while emphasizing the need for future studies including larger populations and multi-seasonal observations.

In summary, our findings highlight the critical role of coordinated wood and leaf traits in shaping sex-specific hydraulic strategies in dioecious species. Integrating anatomical and physiological traits across organs provides a more accurate understanding of plant resilience and vulnerability under climate change. As environmental conditions become increasingly arid, considering sex-based trait coordination will be essential for predicting population dynamics and guiding conservation strategies for Mediterranean shrublands and other dioecious systems.

## Supplementary Material

Supplementary_REV_tpaf133

## Data Availability

The data supporting the findings of this study are available from the corresponding author upon reasonable request.
