# Supplementary material for: Sex matters: understanding wood–leaf hydraulic coordination in dioecious species in a drying world
Source: Tree Physiol. 2025 Oct 22;45(12):tpaf133. doi: 10.1093/treephys/tpaf133 (PMC12684968; doi:10.1093/treephys/tpaf133)
Supplement: Supplementary_REV_tpaf133 [file supplementary_rev_tpaf133.docx]

**Supplementary**

**Table S1.**Results of simple linear regressions between anatomical and physiological traits in *Pistacia lentiscus* and *Rhamnus alaternus*, performed separately for male and female individuals. The table reports the coefficient of determination (R²), the associated p-value, and the statistical significance based on the following thresholds: ns = not significant, * p ≤ 0.05, ** p ≤ 0.01, *** p ≤ 0.001. Trait codes indicate the dependent and independent variables (e.g., AN_SLA: net photosynthesis vs. specific leaf area).

| Specie | Sex | Trait | R² | p-value | Significance |
| --- | --- | --- | --- | --- | --- |
| *P. lentiscus* | male | AN_SLA | 0.805 | 8.32e-08 | *** |
| *P. lentiscus* | male | AN_SD | 0.84 | 1.41e-08 | *** |
| *P. lentiscus* | male | AN_IS | 0.803 | 9.03e-08 | *** |
| *P. lentiscus* | male | FVFM_TT | 0.881 | 6.13e-07 | *** |
| *P. lentiscus* | male | AN_VF | 0.694 | 5.11e-06 | *** |
| *P. lentiscus* | male | TT_VF | 0.843 | 8.31e-07 | *** |
| *P. lentiscus* | female | AN_SLA | 0.217 | 0.0384 | * |
| *P. lentiscus* | female | AN_SD | 0.171 | 0.0698 | ns |
| *P. lentiscus* | female | AN_IS | 0.095 | 0.185 | ns |
| *P. lentiscus* | female | FVFM_TT | 0.186 | 0.0577 | ns |
| *P. lentiscus* | female | AN_VF | 0.117 | 0.141 | ns |
| *P. lentiscus* | female | TT_VF | 0.088 | 0.203 | ns |
| *R. alaternus* | male | AN_SLA | 0.022 | 0.812 | ns |
| *R. alaternus* | male | AN_SD | 0.918 | 3.18e-11 | *** |
| *R. alaternus* | male | AN_IS | 0.947 | 6.16e-13 | *** |
| *R. alaternus* | male | FVFM_TT | 0.67 | 1.04e-05 | *** |
| *R. alaternus* | male | AN_VF | 0.981 | 7.6e-17 | *** |
| *R. alaternus* | male | TT_VF | 0.879 | 1.1e-09 | *** |
| *R. alaternus* | female | AN_SLA | 0.111 | 0.9 | ns |
| *R. alaternus* | female | AN_SD | 0.519 | 0.00239 | ** |
| *R. alaternus* | female | AN_IS | 0.006 | 0.292 | ns |
| *R. alaternus* | female | FVFM_TT | 0.041 | 0.001 | ns |
| *R. alaternus* | female | AN_VF | 0.471 | 0.000837 | *** |
| *R. alaternus* | female | TT_VF | 0.001 | 0.964 | ns |
